# The Action of Ammonia on the Teeth

**Published:** 1882-06

**Authors:** 


### THE ACTION OF AMMONIA ON THE TEETH.

Dr. Geo. Watt has an excellent article on this subject in *The Ohio State Journal of Dental Science*, of which we give the following abstract :

To get a clear idea of the mischief ammonia may do in the mouth we would gain by thinking, for a moment, of the character and composition of the buccal fluids. When normal, the saliva is able to hold all its constituent salts in solution. Its ability to do this depends on the simple fact that it also holds free carbonic acid in solution. Carbonic acid is ordinarily a gas, inodorous, invisible, and nearly tasteless. But when dissolved in water or saliva it is liquid. When liquefied thus, or by any other process, it is readily induced to resume its gaseous taste.

A solution of free carbonic acid is able to hold in solution the subphosphate of lime, called "bone phosphate," and it is precipitated from its solutions just as is carbonate of lime, and under the same circumstances.

It matters not by what process the free carbonic acid is taken from the fluid holding these lime salts in solution, they are precipitated all the same. For example, if the acid is removed by combination with an alkali, the lime salts are precipitated. Dentists are often disappointed to find their patients annoyed by deposits of tartar, even when they are diligently using alkaline dentifrices which they have prescribed. The explanation is that the alkali combines with and neutralizes the free carbonic acid in the buccal fluids, rendering them totally unable to hold the salts in solution, and they are deposited as tartar.

When the constitution is sound and healthy, when each organ is able to, and does, perform its function, the saliva retains its natural amount of free carbonic acid, and hence holds in solution the normal quantity of each lime salt, and no tartar can be deposited. But let the constitution become enfeebled, till the effete matter becomes too abundant for the excretory organs to carry it out of the system, physiology becomes pathology, and organs intended only for secretion, take on excretory work ; the salivary glands, or the mucous follicles, or both, give off ammonia, the free carbonic acid in the saliva unites with it, forming carbonate of ammonia, and the saliva is thus rendered totally unable to dissolve the two lime salts, and they, with epithelial matter, and deteriorated mucus, are precipitated on the teeth in the form of salivary calculus, or tartar. And now we are able to see whether the disease is constitutional, or merely local. If only local, of course, appropriate topical treatment is all that is necessary to its successful management; and there is justification for those who claim to know all about the disease, yet rely, solely, on a thorough scraping for its cure.

A fertile source of ammonia within the mouth may yet be noticed. The organic matter entangled in the lime salts, and thus constituting a portion of tartar, decomposes, yielding the ordinary products of putrefaction, and consequently ammonia. This fact alone calls for the thorough scraping or cleansing universally recommended and practised by all who are conscientious and industrious. Other reasons call for the same treatment, but the one reason is sufficient. To get rid of the ammoniacal odors of our stables, we scrape and shovel out the manure; but if the horse is declining, we scarcely rest content with this alone. The physician who is treating zymotic disease—typhoid, for example—would be ridiculed if he should rely on the removal of the excrements as the only means of cure; and he would be rightfully censured if he should allow them to remain in the sick room during treatment.

It would appear, then, that we have no more right to regard and treat this morbid condition as local, than if were erysipelas, scurvy, scrofula, or consumption. Nor will it do to rely wholly on either local or general treatment, but a legitimate use of both is the proper course.

As to the topical treatment, it has been already intimated that the most thorough removal of all deposits about the necks of the teeth, and especially between the free margins of the gums and the teeth, is necessary. All tartar must be removed, and the teeth must be polished. And if parts of the margins of the gums have so far lost their vitality that normal, healthy circulation cannot be re-established in them, they should also be removed. And the same teaching applies with equal force, to the borders of the sockets, if they too have lost vitality. There is not much danger of overdoing in this direction. Some cases require many sittings, with patience and perseverance on the part of both patient and practitioner. It is not in accordance with the plan of this paper to suggest instruments, or describe manipulations. Let the instruments be the best possible in quality and adaptability, while judgment and skill must guide the operator and control his manipulations.

The mouth having been thoroughly cleansed, it follows to make effort to restore the lost tone to the vessels and fibres of the tissues involved. Tonics and astringents, with friction, may be used, the friction being the most important of these suggestions. Almost any wash may be suggested that necessitates friction in its application. Friction with the tooth brush is not usually sufficient. The morbid blood is not thereby forced out of the loaded vessels. Firm and persevering friction with the finger, over all accessible portions of the gums should be resorted to at least three times a day. The superficial vessels are thus emptied, and the expelled blood does not directly return to them, but that which is fresher and more nutritive takes its place. This already detailed, or other topical treatment to accomplish the same results, is by many regarded as quite sufficient; and with this much done, in many cases nature will be aroused to accomplish the rest. But in aggravated cases, the patient will soon be found as badly off as before, if nothing more is done. If the constitution is still in such a state of deterioration that the mucous follicles of the air passages and the salivary glands are called on to aid in the elimination of ammonia, the saliva cannot hold the lime salts in solution, and tartar must be deposited. The indication is therefore, to get rid of the surplus ammonia. But how?

Bearing in mind that ammonia is a result of the putrefaction of dead, or effete nitrogenous tissue, we must obviate the tendency to death. The weakened organs of the constitution must be restored to their normal power. The excretory organs, if torpid, must be aroused to do their whole duty, so that those whose duty is secretion shall not be called on to excrete. And fortunately, in many cases, the remedies needed as tonics and alteratives for weakened and depraved organs, are such as at the same time neutralize the excess of the objectionable alkali. It is well known that the mineral acids are popular remedies in indigestion where the fault is with the liver or stomach, or even with both. While they stimulate the coats of the stomach to a proper secretion of mucus, and the normal supply of the gastric juice, they also stimulate the liver to an increased secretion of bile, and thus relieve obstruction in the portal circulation, and the consequent congestion of the abdominal and pelvic organs, and at the same time they neutralize the excess of ammonia in the circulation (thus saving the red corpuscles from solution), forming with it highly soluble neutral salts, nearly or quite harmless in the blood, yet easily eliminated therefrom by the emunctories. For such purpose the aromatic sulphuric acid, called elixir vitriol, is often administered. It may be given in doses of ten to thirty drops three or four times a day, diluted with water. Or nitro-muriatic acid, called *aqua regia*, may be used for the same purpose, and in the same way, but in doses of three or four drops. Better results, however, may be obtained from *aqua regia* by using it as a bath. Officinal *aqua regia* is prepared by mixing one part of nitric, with two parts, by measure, of hydrochloric acid ; and in preparing the bath, four to six ounces of it may be added to three gallons of water. The feet may be immersed in the bath, or the liquid may be applied to the surface of the body by a sponge.

If nitric acid is preferred, the officinal dilute acid may be used as directed for elixir vitriol ; and the same direction will answer for hydrochloric acid, but when this is needed it is common to prescribe tincture of iron, which has in it an excess of the acid ; and the iron aids in restoring the red corpuscles to replace those destroyed by the ammonia. This may be given three or four times a day, in doses of ten to twenty drops.

Relief often may be obtained by a free use of vegetable acids. Acetic acid is universally used, and often in spite of strong prejudice. The growing girl drinks vinegar on the sly, and steals pickles and eats them between meals, almost without chewing, and if she suffers from indigestion her case is cited in proof that pickles are not wholesome food. Sometimes we find the prejudice against vinegar so strong, on the part of parents and proprietors of boarding schools, that we have to take advantage of a Latin prescription in order to have it used by the patient. Perhaps no organic acid meets the demand better than acetic, in the class of cases under consideration. Fortunately it is cheap and abundant. But for a patient in fashionable life, it is sometimes better to prescribe citric acid in the shape of lemons, or lemonade. The additional cost sometimes insures attention, and the taste is perhaps more pleasant. These acids may be used almost at will by the patient, but in general should be used only with the regular meals, or with lunch at a definite hour. The stomach should be taxed with nothing but water between meals. This liquid needs no digestion, but passes unchanged into the blood. Bearing this in mind will aid in recalling the principle that should guide in dietary affairs, that anything requiring digestion as a prelude to assimilation, should be taken only at regular meals. We are thus minute because proper nutrition is necessary to the prevention or cure of the morbid state we have been discussing.

Acid fruits often afford relief, and of these the grape is, perhaps, best of all. Strawberries are excellent. Apples, currants, cherries, answer a good purpose, as in many cases they not only neutralize an excess of ammonia already formed, but they prevent excessive formations of it, by imparting a healthy tone to the digestive apparatus, and thus building up the constitution to a state of vigor incompatible with the excessive formations of the objectionable alkali.

